# Determinants of poor utilization of antenatal care services among recently delivered women in Rwanda; a population based study

**DOI:** 10.1186/s12884-017-1328-2

**Published:** 2017-05-15

**Authors:** Akashi Andrew Rurangirwa, Ingrid Mogren, Laetitia Nyirazinyoye, Joseph Ntaganira, Gunilla Krantz

**Affiliations:** 1 Department of Epidemiology and Biostatistics, School of Public Health, University of Rwanda, Kigali, Rwanda; 2 Section of Epidemiology and Social, Medicine (EPSO), Department of Public Health and Community Medicine, The Sahlgrenska Academy at University of Gothenburg, Box 453, 405 30 Göteborg, Sweden; 3 Department of Clinical Sciences, Obstetrics and Gynecology, Umea University, Umea, Sweden; 4 Department of Community Health, School of Public Health, University of Rwanda, Kigali, Rwanda

**Keywords:** Antenatal care utilization, Recently delivered women, Rwanda

## Abstract

**Background:**

In Rwanda, a majority of pregnant women visit antenatal care (ANC) services, however not to the extent that is recommended. Association between socio-demographic or psychosocial factors and poor utilization of antenatal care services (≤2 visits during the course of pregnancy irrespective of the timing) among recently pregnant women in Rwanda were investigated.

**Methods:**

This population-based, cross sectional study included 921 women who gave birth within the past 13 months. Data was obtained using an interviewer-administered questionnaire. For the analyses, bi-and multivariable logistic regression was used and odds ratios were presented with their 95% confidence intervals.

**Results:**

About 54% of pregnant women did not make the recommended four visits to ANC during pregnancy. The risk of poor utilization of ANC services was higher among women aged 31 years or older (AO*R*, 1.78; 95% CI: 1.14, 2.78), among single women (AOR, 2.99; 95% CI: 1.83, 4.75) and women with poor social support (AOR, 1.71; 95% CI: 1.09, 2.67). No significant associations were found for school attendance or household assets (proxy for socio-economic status) with poor utilization of ANC services.

**Conclusion:**

Older age, being single, divorced or widowed and poor social support were associated with poor utilization of ANC services. General awareness in communities should be raised on the importance of the number and timing of ANC visits. ANC clinics should further be easier to access, transport should be available, costs minimized and opening hours may be extended to facilitate visits for pregnant women.

## Background

Antenatal care (ANC) is an important determinant of maternal and perinatal mortality and ANC attendance is an essential component of maternal health care on which the health of mothers and newborns depend [1]. Globally, the number of women dying due to complications during pregnancy and childbirth decreased by nearly 50% from 1990 to 2013 but the number of deaths remains unacceptably high especially in low-income countries where 99% of these deaths occur [2, 3]. Within the East Africa community member states, maternal mortality ratios are among the highest on the continent at 740, 410, and 400 deaths per 100,000 live births in Burundi, Tanzania and Kenya, respectively [2]. The lifetime risk of maternal mortality due to pregnancy complications, such as obstructed labour, excessive bleedings or eclampsia, is higher in Sub-Saharan Africa than in other parts of the world in part due to higher fertility rates, poorer nutritional status of women, inaccessibility to ANC clinics and higher prevalence of vulnerable life circumstances such as poverty and a higher prevalence of HIV infection [2, 4]. Also, higher ANC attendance among pregnant adolescents is particularly important as complications during pregnancy and childbirth have been shown to be a major cause of death among girls aged 15–19 in low- and middle-income countries.[2] Rwanda is a small country in the center of the African continent with approximately 11 million inhabitants. Sixty-four percent of women and 66% of men in Rwanda have completed or received some primary school education [5]. However, 12% of women and nine percent of men have no formal education [5, 6]. The majority of the low educated or illiterate women live in rural areas where more than 93% of the women are involved in small-scale agricultural activities, and fertility rate is higher than the country average of 4.6 children. Following the 1994 genocide against the Tutsi in Rwanda, the rates of maternal mortality increased, but gradually declined to 340 deaths per 100,000 live births in 2012 [2, 7]. This improvement in maternal health may be related to increase in attendance at ANC services resulting from intensive sensitization of pregnant women by local government authorities and community health workers [6, 8–10]. As in other low and middle-income countries, about 60% of maternal deaths in Rwanda is related to conditions that can be detected and addressed during the antenatal period. Such conditions include hypertension, HIV/AIDS infection, pre-existing medical conditions and sexually transmitted infections. Emergency situations may arise during childbirth, such as obstructed labour, severe bleeding and other problems that require emergency care [7, 11]. Deaths could be avoided if pregnant women have proper access to ANC services during pregnancy and support by a trained midwife during childbirth [3]. Even if trained staff is of main importance, also what procedures that are performed and the equipment available make a difference [12]. Antenatal care services in Rwanda are available at health centers and mainly provided by nurses (94%) [13]. If complications arise, the pregnant mother is referred to a district hospital for further assistance. Previous studies have suggested that demographic, socio-economic and behavioral factors such as age, time and cost of travel to the health facility, big family size and poor access to social support are associated with poor antenatal care attendance [2, 14]. Furthermore, a study on maternal health care across sub-Saharan Africa found that low level of education, rural residence, and low household income were all associated with poor ANC attendance [15]. Although Rwanda has adopted the previous WHO model of antenatal care, which recommends one visit during each trimester of gestation and a final visit immediately preceding delivery for women without pregnancy-related complications or risk factors, still only a proportion of pregnant women make all four recommended visits during pregnancy [5, 7]. This raises concerns regarding the adoption and implementation of the WHO new guidelines, which recommend that the number of contacts a pregnant woman has with a health provider throughout her pregnancy should increase from four to eight [16].

The aim of this population-based study was therefore to investigate number and timing of ANC visits that were performed, and socio-demographic and psychosocial risk factors for low or no attendance.

This study forms part of the Maternal Health Research Programme (MaTHeR) undertaken by the University of Rwanda in collaboration with University of Gothenburg and Umeå University in Sweden.

## Methods

### Study design, study population and sample size

This cross-sectional population based study was conducted in the Northern province and in Kigali city, the capital and largest city in Rwanda. Kigali city has urban, semi-urban and rural areas, whereas the Northern Province is predominantly rural. The target population was women who gave birth within the past 13 months. The selection process was based on the total population of about 2,865,000 inhabitants from 4 791 villages.[13]. The sample size was calculated based on the estimated prevalence of hypertensive disorders during pregnancy (10%) [17–19], as hypertension is one of the major factors to be investigated within this research programme, and was the least prevalent among study outcomes. The desired level of precision was set at 0.025 and considering the effect of multi-stage sampling; a design effect of 1.5 was used. Adding 10% to the sample size to take care of possible non-responses, the total sample size was calculated to be 922 women. The selection process was based on a total population of about 2,865,000 inhabitants from 4 791 villages [13]. In three steps, villages (the smallest administrative entity in Rwanda), households and study participants were randomly selected in the five districts of the Northern Province and in three districts within Kigali City. Firstly, out of 4 791 villages, it was decided to select in total 48 villages (equal to 1%). The villages were then randomly selected proportionate to total number of villages in each district by using Epi-Info random function. Approximately 20% of Rwandan population lives in urban areas [6]. In order to mirror the country’s rural-urban divide, 20% of the villages were selected from urban areas. Secondly, households from each village were selected based on the total number of the households in each selected village (proportionate to size). With the help of community health workers who keep records of maternal pregnancies and childbirths, women who gave birth within the past 13 months were identified, and finally the women to be interviewed were randomly selected among eligible women in each household, if more than one were present. In case of no eligible woman in the household, the closest household with an eligible woman was approached. If an excess number of households with an eligible woman were at hand in a village, lottery decided which ones to include. In case of fewer eligible women in the village than envisaged in the study, the closest village was approached and the same data collection procedures were used to obtain the remaining number of eligible women. All selected women but one gave their consent to participate (overall response rate was 99.9%.), and some internal non-responses were at hand.

### Data collection procedures

Data collection took place between July and August of 2014. A structured questionnaire was developed, which included questions relating to ANC attendance and procedures, delivery procedures, pregnancy and delivery complications and cost of ANC services. The questionnaire was translated into Kinyarwanda, the Rwandan national language and pre-tested but no major changes were made on the questionnaire after the pilot study apart from a few adjustments in wording. Twelve well-trained interviewers who were all women belonging to a pool of interviewers at the School of Public Health, University of Rwanda were selected. These were nurses, midwives and clinical psychologists. Face-to-face interviews were performed and four supervisors (first author and three colleagues) guided the interviewers. The supervisors ensured that all selected households were contacted and reviewed the questionnaires before the team left the village. For the protection of the interviewed women in the households and for confidentiality purposes, only one person in each household was interviewed. The School of Public Health, College of Medicine and Health Sciences, University of Rwanda, was the lead implementer of the study. Data entry was performed by four skilled personnel selected from a permanent cohort of data entry clerks from the School of Public Health under the supervision of a data entry manager.

### Variables

#### Utilization of antenatal care services

Number and timing of antenatal care visits was used as the dependent variable and two different variables were tried in the initial analyses. The first one used ≤2 visits at ANC clinic during the course of pregnancy irrespective of the timing as the poor ANC services utilization. For comparison reasons, the second one used ≤2 visits plus those who made 3 visits but none during first trimester as the poor ANC services utilization.

#### Socio-demographic and psychosocial factors

*Participants’ age* was categorized into 15–30 years and 31–46 years age groups*. The number of people in the household* was described as a three-category variable (1–3 people, 4–6 people and 7 or more) then, a dichotomized variable was created where the first two categories were combined and the latter one considered the exposed. *Marital status* was dichotomized into married or cohabitating and then single, divorced or widowed were brought together in the exposure category. *Women’s relationship with household head* was assessed as being the wife, daughter, daughter in law, other family relationship and no relationship, further dichotomized into the wife or any other relationship as the exposure category. *Ever attended school* was responded with yes/no with the latter as the exposure category. *Total household monthly income* was made into a three-category variable as more than 36,000 FRW (60USD), between 17,501 and 35,999 FRW (30–60 USD) and less than 17,500 FRW (30 USD) later dichotomized into ≤17, 500FRW and ≥17, 501FRW. *Social support* was defined as having a family member, a relative or a friend who could lend support to the woman if any problem would arise. This item was responded to with yes/no, with the latter as the exposure category. *Partner’s age* was categorized into ≤ 40 and 41–70 age groups. Then, identical techniques were used to categorize partner’s level of education and the total household monthly income as described above for participants. *A composite variable of assets in the household* was used as a proxy for socio-economic status of the household. Assets in the household included a radio, a television set, a refrigerator, a bicycle, a motorcycle, a car, a mobile phone and a computer. It was later dichotomized into having at least one of the items or having none of the items. Having none of the items constituted the very poor subjects.

### Statistical analysis

Frequency and prevalence (n and %) were used to describe participants’ characteristics. Two variables were tried as dependent variables, i.e., poor ANC utilization, ≤2 visits to ANC (*n* = 122) irrespective of when in the course of the pregnancy, and a composite variable of those with ≤2visits plus those with 3 visits but none in the 1st trimester (*n* = 317). Associations between socio-demographic and psychosocial factors and poor utilization of antenatal care services were calculated by use of bi- and multivariable logistic regression models. As the results were rather similar for the two dependent variables in the bivariate analyses, it was decided to use the strongest criteria for poor ANC attendance (≤2 visits) in the further analysis to identify the groups of women most at risk of low ANC attendance. Those socio-demographic and psychosocial factors that displayed statistical significance in the bivariate analyses were entered into the regression model in a stepwise fashion. For theoretical reasons, *ever-attended school* and *assets in the household* (proxy for socio-economic status) were further tried in the multivariable analyses, as these have been important predictors in other studies. If two variables were highly correlated (*r* ≥ 0.40), one was excluded. Using this approach, pregnant women’s relationship with household head, the sex of the household head and husband’s age were not included in the final model as these variables were highly correlated with marital status. Also, parity and number of previous pregnancies were excluded because they were highly correlated with the number of people in the household*.* The fit of the final model was assessed by using Nagelkerke R-Square test. Finally, potential interactions between variables in the final model were tested but no statistically significant interactions were present. All measures of association are presented as odds ratios (OR) with their 95% confidence interval (95% CI). All the analyses were performed using Statistical Package of Social Sciences version 22.0 for windows (SPSS, Armonk, NY, USA).

## Results

### Socio-demographic and psychosocial characteristics

Participants were mostly of low socio-economic status, i.e., poor, had not completed primary school and were engaged in non-skilled work (Table 1). The majority of the women were married or cohabiting (84.1%, *n* = 774) and 20.2% (*n* = 186) of the respondents had no family member, relative or friend who could lend support if any problem would arise. Partners’ socio-demographic and psychosocial characteristics showed a similar trend; a bigger proportion had not completed primary school and were mostly involved in non-skilled work; 42.9% (*n* = 283) and 44.5% (*n* = 343), respectively (Table 1).

**Table 1:** Socio-demographic and psychosocial characteristics of study population by number and timing of antenatal care visits (*n* = 921)

Characteristics	Total n (%)	≤2visits n (%)	≥3visits n (%)	*p*-value	≤2visits^a^ n (%)	≥4visits^b^ n (%)	*p*-value
Age groups (*n* = 919)							
15–30	632(68.8)	72(59.0)	560(70.3)	0.01	194(61.2)	438(72.8)	0.0001
31–46	287(31.2)	50(41.0)	237(29.7)		123(38.8)	164(27.2)	
Number of people in the household (*n* = 917)							
0–6	748(81.6)	86(70.5)	662(83.3)	0.001	244(77.2)	504(83.9)	0.01
7 or more	169(18.4)	36(29.5)	133(16.7)		72(22.8)	97(16.1)	
Relationship with household head (*n* = 919)							
Not wife	166(18.1)	42(34.4)	124(15.6)	0.0001	74(33.3)	92 (15.3)	0.003
Wife	753(81.9)	80(65.6)	673(84.4)		243(76.7)	510(84.7)	
Sex of the house hold head (*n* = 914)							
Female	70(7.7)	17(14.0)	53(6.7)	0.005	33(10.5)	37(6.2)	0.02
Male	844(92.3)	104(86.0)	740(93.3)		282(89.5)	562(93.8)	
Marital status (*n* = 919)							
Single, divorced, widowed, separated	145(15.8)	37(30.3)	108(13.6)	0.0001	69(21.8)	76(12.6)	0.0001
Married or cohabitating	774(84.2)	85(69.7)	689(86.4)		248(78.2)	526(87.4)	
Ever attended school (*n* = 913)							
Yes	829(90.8)	105(86.1)	724(91.5)	0.05	285(90.2)	544(91.1)	0.64
No	84(9.2)	17(13.9)	67(8.5)		31(9.8)	53(8.9)	
Highest attained level of education (*n* = 830)							
Incomplete primary school	416(50.1)	59(55.7)	357(49.3)		167(58.2)	249(45.9)	
Complete primary school or vocational training	219(26.4)	21(19.8)	198(27.3)	0.24	64(22.3)	155(28.5)	0.003
Secondary school or university	195(23.5)	26(24.5)	169(23.3)		56(19.5)	139(25.6)	
Occupation (*n* = 907)							
Skilled work, civil servant, student	119(13.1)	16(13.2)	103(13.1)		40(12.8)	79(13.3)	
Non skilled work	528(58.2)	66(54.5)	462(58.8)	0.62	186(59.6)	342(57.5)	0.82
Not employed, other occupation	260(28.7)	39(32.2)	221(28.1)		86(27.6)	174(29.2)	
Social support (*n* = 919)							
Yes	733(79.8)	87(71.3)	646(81.1)	0.01	253(79.8)	480(79.7)	0.97
No	186(20.2)	35(28.7)	151(18.9)		64(20.2)	122(20.3)	
Partner Characteristics							
Husband age (*n* = 775)							
≤ 40	667(86.1)	61(72.6)	606(87.7)	0.0001	196(78.1)	471(89.9)	0.0001
41–70	108(13.9)	23(27.4)	85(12.3)		55 (21.9)	53(10.1)	
Husband ever attended school(*n* = 766)							
Yes	667(87.0)	69(84.1)	598(87.3)	0.42	212(85.1)	455(87.8)	0.29
No	100(13.0)	13(15.9)	87(12.7)		37(14.9)	63(12.2)	
Highest level of education (*n* = 659)							
Incomplete primary school	283(42.9)	31(45.6)	252(42.6)		91(42.9)	192(43.0)	
Complete primary school or vocational training	248(37.6)	28(41.2)	220(37.2)	0.32	92(43.4)	156(34.9)	0.02
Secondary school or university	128(19.4)	9(13.2)	119(20.1)		29(13.7)	99(22.1)	
Husband’s occupation (*n* = 770)							
Skilled work, civil servant, student	174(22.6)	14(17.1)	160(23.3)		55(22.2)	119(22.8)	
Non skilled work	343(44.5)	33(40.2)	310(45.1)	0.11	122(49.2)	221(42.3)	0.15
Not employed or other occupation	253(32.9) 253	35(42.7)	218(31.7)		71(28.6)	182(34.9)	
Total household monthly income (*n* = 861)							
< 17500FRW	258(30.0)	35(31.3)	223(29.8)		103(35.0)	155(27.3)	
17501-35999FRW	240(28.0)	24(21.4)	216(28.8)	0.24	75(25.5)	165(29.1)	0.06
36000 or more FRW	363(42.0)	53(47.3)	310(41.4)		116(39.5)	247(43.6)	
Household assets summary measure (*n* = 909)							
Improved (≥1 item)	715(78.7)	91(74.6)	624(79.3)	0.23	247(78.7)	468(78.7)	0.99
Poor (none of the items)	194(21.3)	31(25.4)	163(20.7)		67(21.3)	127(21.3)	

*P*-value are from Chi-square test and show the relationship between categorical predictor variables and the first outcome variable (≤2 visits and ≥3visits) and the second outcome variable (≤2visits^a^ and ≥4visits^b^)

^a^ ≤ 2 visits plus 3 visits but none during the 1st trimester

^b^ ≥ 4visits plus those with 3 visits of which at least one was done in the first trimester

### Pregnancy related characteristics of the study population

First time pregnant women constituted 32.4% (*n* = 288) of the participants. The majority of women visited health centres for ANC services and were mostly seen by nurses and a few by a trained midwife. A considerable proportion of the women, (30.9%, *n* = 276) had to walk for one hour or more to seek ANC services. The women who made the recommended four or more visits to ANC during pregnancy constituted 45.6% (*n* = 418) of the participants while 13.3%, (*n* = 122) made two or less visits. Thirty four percent (*n* = 317) of the participants were in the group composed of those with <2visits plus those with 3 visits but none in the 1st trimester. The majority of the women delivered the child at a health centre (*n* = 582, 63.3%), while 5.1% (*n* = 47) delivered at home or while on their way to a health facility. To our surprise, 81.6% (*n* = 746) of the cases, the husband accompanied the pregnant woman to the first ANC visit but 104 (11.4%) women walked to the health facility alone (Table 2).

**Table 2:** Pregnancy related characteristics of the study population (*n* = 921)

Variables	*n*	%
Pregnancies before the latest one (*n* = 889)		
First time pregnancy	288	32.4
≥ 2 pregnancies	601	67.6
Type of ANC facility visited (*n* = 915)		
Health center or dispensary	887	97.0
District hospital	12	1.3
Referral hospital	7	0.8
Private clinic	8	0.9
Other	1	0.1
Responsible health care professional during first visit (*n* = 896)		
Nurse/midwife	822	91.3
Doctor	74	8.7
Who accompanied pregnant woman during first visit to ANC clinic (*n* = 914)		
Husband	746	81.6
Mother, family member or any other person	19	2.1
Community Health Worker	45	4.9
None	104	11.4
Time walking to ANC clinic (*n* = 892)		
5–30 min	349	39.1
31–60 min	267	29.9
1–2 h	195	21.8
2–3 h	68	7.6
> 3 h	13	1.5
Place of delivery (*n* = 919)		
At home or on the way to a health facility	47	5.1
Health center or dispensary	582	63.3
District hospital	230	25.0
Referral hospital	50	5.4
Private clinic	10	1,1
Did you attend ANC clinic during pregnancy?		
Yes	910	99.5
No	5	0.5
Number of Antenatal care visits (*n* = 915)		
1 visit	23	2.5
2 visits	94	10.3
3 visits	380	41.6
4 visits	380	41.6
≥ 5 visits	38	4.0
Antenatal care (ANC) visits (*n* = 920)		
≤ 2 visits	122	13.3
≥ 3 visits	798	86.7
Antenatal care (ANC) visits (*n* = 920)		
≤ 2 Visits + 3 visits but none during first trimester of pregnancy	317	34.5
≥ 3 visits	603	65.5

### Risk factors of poor utilization of antenatal care

Bivariate analyses showed that women from higher age groups, from households with large number of people and women who were family heads were at higher risk of poor utilization of ANC services, defined as making two visits or less during the entire pregnancy (Table 3). Also, women who were not married to the household head, or who were single, divorced, widowed or separated made fewer ANC visits, with an odds ratio of 2.85 (95% CI: 1.87, 4.33 and OR 2.77 (95% CI: 1.79, 4.29), respectively. Being married to a husband aged 41 years or more was highly associated with poor utilization of ANC services (Table 3). In the multivariable analyses the risk of poor utilization of ANC services was almost twice as high in the older age group as compared to women aged 30 years or less, with OR of 1.78 (95% CI, 1.14, 2.78). The variable number of people in the household lost its statistical significance when marital status was entered into the model. Further, it was found that women who were single, divorced, widowed or separated were at a higher risk of poor utilization of ANC services as compared to married women, (OR) 2.99 (95% CI: 1.83, 4.75). Also, those who had no family, relative or friend to support them when in need were at a higher risk of poor utilization of ANC services as compared to women who had support (OR 1.71; 95% CI 1.09, 2.67). No schooling and being poor, with no assets in the household, were not associated with poor ANC attendance (Table 4).

**Table 3:** Associations between socio-demographic and psychosocial characteristics and poor utilization of ANC services (*n* = 921)^a^

	n (%) with poor ANC utilization	OR^b^	95% CI
Age groups (years)			
15–30	72 (11.4)	1	
31–46	50 (17.4)	1.64	1.11–2.42
Number of people in the household			
1–6	86(11.5)	1	
≥ 7	36 (21.3)	2.08	1.35–3.21
Pregnancies before the latest one			
No pregnancy before	29 (10.1)	1	
One or more pregnancies before	87 (14.5)	1.51	0.96–2.35
Sex of the household head			
Male	104 (12.3)	1	
Female	17 (24.3)	2.28	1.27–4.09
Relation with household head			
Wife	80 (10.6)	1	
Relationship other than wife	42 (25.3)	2.85	1.87–4.33
Marital status			
Married or cohabitating	85 (11.0)	1	
Single, divorced, widowed, separated	37(25.5)	2.77	1.79–4.29
Ever attended school			
Yes	105 (12.7)	1	
No	17 (20.2)	1.75	0.98–3.09
Social support			
Yes	87 (11.9)	1	
No	35 (18.8)	1.72	1.12–2.65
Time walking to ANC clinic			
1–60 min	76 (12.3)	1	
> 1 h	35 (12.7)	1.03	0.67–1.58
Husband’s age (years)			
≤ 40	61(9.1)	1	
41–70	23 (21.3)	2.69	1.58–4.57
Household assets summary measure			
Improved (≥1 item)	91 (12.7)	1	
Poor (none of the items)	31 (16.0)	1.30	0.83–2.03

^a^Poor utilization of ANC services defined as ≤2visits during pregnancy

^b^Values are crude odds ratio and 95% confidence intervals that indicate the differences in risks of poor utilization of ANC services compared to reference category (

**Table 4:** Associations between socio-demographic and psychosocial characteristics and poor utilization of antenatal care services (*n* = 921)^a^

Variable	Poor utilization of antenatal care service^b^					
	Model 1	Model 2	Model 3	Model 4	Model 5	Model 6^c^
Age groups						
15–30	1					
31–46	1.64(1.11, 2.42)	1.35(0.88, 2.06)	1.82(1.17, 2.83)	1.80(1.15, 2.80)	1.76(1.13, 2.27)	1.78(1.14, 2.78)
Number of people in the household						
1–6		1				
7 or more		1.84(1.16, 2.93)	1.45(0.91,2.31)	1.45(0.91, 2.31)	1.49(1.01, 2.39)	1.50(0.93, 2.40)
Marital status						
Married or cohabitating			1			
Single, divorced, widowed, separated			3.06(1.92, 4.89)	3.06(1.92, 4.90)	3.04(1.89, 4.86)	2.99(1.83, 4.75)
Ever attended school						
Yes				1		
No				1.69(0.94, 3.03)	1.63(0.90, 2.92)	1.55(0.85, 2.84)
Social support						
Yes					1	
No					1.73(1.11, 2.69)	1.71(1.09, 2.67)
Assets in household, summary measure						
Improved (≥1 higher standard item)						1
Poor (none of higher standard item)						1.12 (0.69, 1.79)

^a^Significant variables were entered into the model one by one. Values are adjusted odds ratio and their 95% confidence intervals from multivariable logistic regression models that indicate the differences in risks of poor utilization of ANC services compared to reference category (1)

^b^Poor utilization of ANC services defined as ≤2visits during pregnancy

^c^Final model with adjusted odds rations and their 95% confidence interval. Variables in the same model are adjusted for each other and for woman and husband’s occupation

## Discussion

Even though maternal mortality has decreased over the years and increasing number women do use ANC services during pregnancy, many women still do not follow the recommendation of four visits or more. We found in this study that only 46% made the recommended four visits or more, while 54% did not, which implies that action is needed to improve the situation. Our findings on risk factors associated with fewer ANC visits than recommended are consistent with findings in other similar studies in Sub-Saharan Africa. A community based cross-sectional study in Ethiopia shows that 42% of study participants made less than four visits to ANC clinics during pregnancy [20]. Similar findings were found in a study in Uganda [21]. This study further examined the association between socio-demographic and psychosocial factors and poor utilization of ANC services. Our findings show that women in the higher age group and single/divorced/widowed women were at a higher risk of making two or less visits to ANC services. Furthermore, women with poor social networks, i.e., women who had no family member or close friend to assist when in need, were at risk of poor attendance at ANC. No associations were found of school attendance or household assets, as a proxy for socioeconomic status, with poor utilization of ANC services.

Several factors have been suggested as possible risk factors for poor utilization of ANC services in low and middle-income countries. A previous study among 422 women in Ethiopia shows that pregnant women in the age range of 15–19 years were more likely to seek ANC care services compared to those between 35 and 39 years, [22] which is in line with our findings. Another study in Tanzania investigated factors associated with ANC visits and found that younger women had significantly higher odds of making four ANC visits as compared to women in older age groups [23]. However, studies investigating the association of women’s age with ANC utilization are not consistent [23, 24]. A study assessing the factors related to utilization of ANC services in Vietnam demonstrated that age did not affect its utilization [24]. Our findings could be explained by a greater confidence and gained experience from earlier pregnancies among older women; they are more likely to have more children, and women who give birth to their first child without any complication tend not to visit health centres for ANC during subsequent pregnancies [25].

We further found that women, who were single, divorced or widowed and women who had no support from family, relative or friend were at a high risk of poor utilization of ANC services. Many studies show similar findings [26–29]. The relationship between marital status and ANC utilization has been inconclusive. A survey of 2199 respondents in Nigeria showed that married women were less likely to seek antenatal care services than their single counterparts [30]. One of the reasons that have been suggested is that married or cohabitating women are not financially independent and might have to seek permission from their spouses and partners to visit ANC services [30]. The reason for married women to make more visits to ANC may be explained by the support married and cohabitating women in Rwanda get from their husbands or partners as a result of ANC attendance sensitization campaign, which equally targets men and encourage them to follow their wife/partner to the clinic. Alternatively, a husband may stay in charge of the household when the wife travels to the ANC clinic. The observation that women with low support had lower odds of using antenatal care as compared to women who had sufficient support is consistent with previous findings [31]. There is greater variation in support among women in Rwanda following war and the 1994 genocide against the Tutsi. Many women lost their parents, siblings, relatives and friends, i.e., their supportive network, and may not have someone to physically stay in charge of the household when they are to leave for the ANC check-up. Also, support from family, a relative or a friend means understanding, sympathy and communication, which can have a positive impact on ANC services attendance.

In contrast to previous studies from low and middle-income countries, which have shown a strong association between school attendance and good socio-economic status respectively, with ANC visits [21, 32–35], we did not find any such association. This might be due to the Government initiative to implement a health insurance scheme, Mutuelles de Santé, or community-based mutual health insurance [10], making ANC visits affordable to a majority of the population and also due to the intensive sensitization and education by community health workers to support women to visit the ANC services.

### Methodological considerations

The strength of this study is the collection of data that was done by well-educated and trained interviewers in face-to face communication. Furthermore, the data collectors were of the same sex and of similar age as the participants, which has been shown to improve the accuracy of the reporting in interviews [36]. The sample size was comparatively large, including 921 women with only one woman refusing to participate. Women from both rural and urban settings were included in proportions equal to what is found in the country and their socio-economic status classified according to previously used standard procedures [6, 37]. However, the cross-sectional nature of our study limits the ability to draw any causal inferences and residual confounding resulting from other socio-demographic and psychosocial related determinants cannot be ruled out. Also, the majority of information inquired about in this study was self-reported, which may have resulted in underreporting of certain determinants. But it is plausible to assume that this was less likely because when the self-reported numbers of visits were checked with patients’ ANC records, similar number of visits was found. Furthermore, data were collected retrospectively from respondents, which may have resulted in recall bias. However, the short recall period of maximum thirteen months meant that this was likely a minor problem. We believe the findings in this study are possible to generalize to the entire country, as living circumstances are quite similar in the four provinces.

## Conclusion

This study found that a majority of women still do not complete the recommended number of four visits to ANC during pregnancy. General awareness in communities should be raised on the importance of number and timing of ANC visits particularly among pregnant women and their husbands. Emphasis should further be on supporting pregnant women of older age, living alone or with a poor social network to follow the recommendations of ANC care. Ministry of Health and health care services, ANC professionals and community health workers have an important task in raising consciousness on this matter. ANC clinics should further be easier to access, transport should be available and opening hours may be extended to facilitate visits for pregnant women and quality of care should be investigated.

## Abbreviations

AIDS: Acquired immune deficiency syndrome
ANC: Antenatal care
AOR: Adjusted odds ratio
DHS: Demographic and Health Survey
FRW: Francs Rwandais
HIV: Human immunodeficiency virus
MaTHeR: Maternal Health Research Programme
NISR: National institute of Statistics of Rwanda
USD: United States dollars
WHO: World Health Organization
